# QuadST identifies cell–cell interaction–changed genes in spatially resolved transcriptomics data

**DOI:** 10.1101/gr.279859.124

**Published:** 2025-08

**Authors:** Xiaoyu Song, Yuqing Shang, Michelle E. Ehrlich, Panos Roussos, Guo-Cheng Yuan, Pei Wang

**Affiliations:** 1 Centre for Quantitative Medicine, Duke-NUS Medical School, Singapore 169857;; 2 Departments of Neurology, Pediatrics, and Genetics and Genomic Sciences, Icahn School of Medicine at Mount Sinai, New York, New York 10029-5674, USA;; 3 Center for Disease Neurogenomics, Department of Psychiatry, Department of Genetics and Genomic Sciences, Friedman Brain Institute, Icahn School of Medicine at Mount Sinai, New York, New York 10029-5674, USA;; 4 Mental Illness Research Education, and Clinical Center (VISN 2 South), James J. Peters VA Medical Center, Bronx, New York 10468, USA;; 5 Department of Genetics and Genomic Sciences, Icahn School of Medicine at Mount Sinai, New York, New York 10029-5674, USA;; 6 Department of Genetics and Genomic Sciences, Tisch Cancer Institute, Icahn School of Medicine at Mount Sinai, New York, New York 10029-5674, USA

## Abstract

Recent advances in spatially resolved transcriptomics (SRT) have provided valuable avenues for identifying cell–cell interactions and their critical roles in diseases. Here, we introduce QuadST, a novel statistical method for the robust and powerful identification of cell–cell interactions and their impacted genes in single-cell SRT. QuadST models interactions at different cell–cell distance quantile levels and innovatively contrasts signals to identify interaction-changed genes, which exhibit stronger signals at shorter distances. Unlike other methods, QuadST does not require the specification of interacting cell pairs. It is also robust against unmeasured confounding factors and measurement errors of the data. Simulation studies demonstrate that QuadST effectively controls the type I error, even in misspecified settings, and significantly improves power over existing methods. Applications of QuadST to real data sets reveal biologically significant interaction-changed genes across various cell types.

Cells rarely function in isolation; instead, they interact with neighboring cells to carry out diverse biological functions. These cell–cell interactions are integral to numerous biological processes (Baszkin and Norde 1999), such as development and immune response. Malfunctions in the cell–cell interactions underlie many complex human diseases, such as cancer (Maffuid and Cao 2023) and neurodegenerative diseases (Szepesi et al. 2018). Spatially resolved transcriptomics (SRT) provides an invaluable avenue for understanding the cell–cell interactions, by allowing researchers to study the gene expression profiles of cells within the native tissue context (Burgess 2019).

Several statistical methods (Arnol et al. 2019; Cang and Nie 2020; Dries et al. 2021; Fischer et al. 2023; Li et al. 2023; Pham et al. 2023; Ma et al. 2024) have been developed for SRT data to identify cell–cell interactions and interaction-changed genes (ICGs), which are the genes whose expression levels are influenced by the spatial proximity of other cells. Among these methods, SVCA (Arnol et al. 2019), SpaOTsc (Cang and Nie 2020), SpatialDM (Li et al. 2023), and stLearn (Pham et al. 2023) analyze interactions by summarizing all cells collectively, regardless of their specific cell types, providing limited insights into cell type–specific interactions. Additionally, SVCA only estimates the proportion of total variations explained by cell–cell interactions without identifying specific genes. SpaOTsc, SpatialDM, and stLearn focus on ligand-receptor pairs, overlooking other interaction mechanisms (e.g., gap junctions) and failing to capture genes involved in the upstream and downstream pathways of the cell–cell interactions.

Meanwhile, RECCIPE (Ma et al. 2024), Giotto (Dries et al. 2021), and NCEM (Fischer et al. 2023) aim at screening the gene expression profiles to identify those involved in cell type–specific cell–cell interactions. RECCIPE is designed for low resolution multicell SRT data, and Giotto and NCEM are for high resolution single-cell SRT data. Unfortunately, Giotto and NCEM require categorizing cells into neighboring interacting pairs versus distant noninteracting pairs, a process prone to errors due to the limited knowledge on the cell type–specific interacting distances. Additionally, current SRT technologies struggle to quantify cell distances accurately, as they cannot accurately capture cell boundaries. These methods often rely on the biased cell center-to-center distance for analysis but lack features to minimize the impact of measurement errors on their identifications. Finally, factors influencing cell–cell interactions in their local microenvironment, such as extracellular elements from a third cell type, are complex and largely unexplored. These factors can confound the analysis, causing inflated false discoveries and reduced power, whereas existing methods cannot address the unmeasured confounders.

We propose QuadST, a highly robust and powerful approach for identifying cell–cell interactions and ICGs on single-cell SRT data. For each cell-type pair, QuadST directly models the measured cell–cell distance as a continuous outcome on gene expression, avoiding the need for cell pair categorization and safeguarding against measurement errors. By innovatively contrasting the associations at different distance quantile levels, QuadST captures enhanced signals from closer cells, providing a strategy for powerful and robust identification of ICGs, even in the presence of inaccurately measured cell–cell distances, unmeasured confounding factors, and gene–gene correlations.

## Results

### QuadST framework

**Figure 1.**
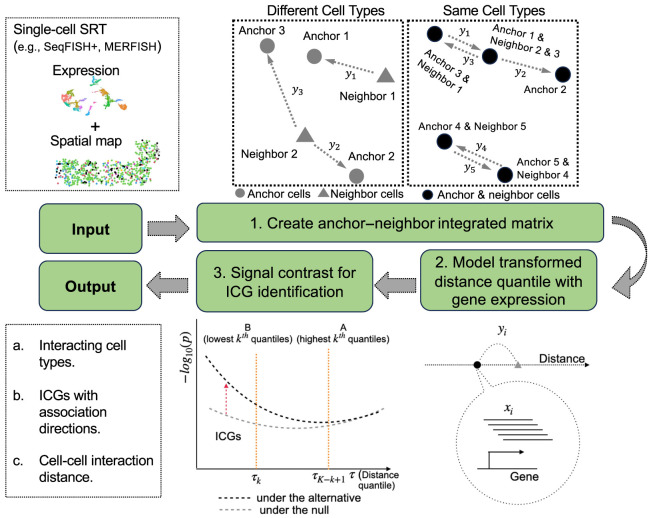
Schematic overview of QuadST. Taking the spatial and transcriptomic data of single-cell SRT as input, QuadST creates an integrated matrix for each anchor–neighbor cell-type pair, models the association between expression and a weighted anchor–neighbor cell–cell distance at different distance quantile levels, and contrasts signal levels at different quantile levels to identify cell–cell interactions and their involved genes with well-controlled FDR.

QuadST has three key steps (Fig. 1; Methods). First, it constructs an integrated matrix for each “anchor” and “neighbor” cell-type pair of analysis. The “anchor” and “neighbor” cells can come either from the same cell type or two different cell types, making a total *K*^2^ cell-type pairs for *K* cell types of interest. The integrated matrix includes the expression profiles and cell-level covariates for all “anchor” cells, along with their distances to their *k* nearest cells in the “neighbor” cell type. Second, QuadST models the association between gene expression and a weighted average of *k* anchor–neighbor cell–cell distances, in parallel for all genes, at a grid of evenly distributed quantile levels of the distance, using quantile regression. By modeling distance as the outcome in quantile regression, we can observe its local associations with gene expression levels, facilitating the comparison of how interactions differ when two cells are close versus far away from each other. Third, QuadST contrasts the signal levels at different distance quantile levels to identify ICGs with false discovery rate (FDR) control. This contrast calculates the ratio of the number of genes with small *P*-values at symmetric upper versus lower quantile levels, such as at above 90^th^ versus below 10^th^ quantile levels, which provides an upper bound for empirical FDR (eFDR), even when *P*-values are ill-behaved, such as arising from regressions with unmeasured confounders and gene–gene correlations.

### Simulation studies

**Figure 2.**
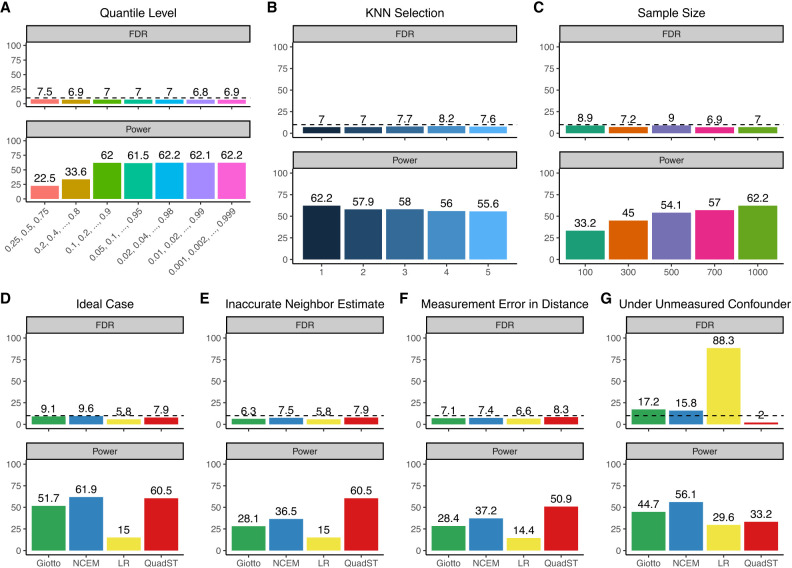
Evaluation of FDR and power for QuadST across different parameters and data sets, and comparison of its performance with alternative methods in various settings. (*A*) FDR and power of QuadST using different number of quantile levels. (*B*) FDR and power of QuadST using different number of *k* nearest neighbors (KNN) at *k* = 1, …, 5. (*C*) FDR and power of QuadST in data with different cell numbers per cell type. (*D*) Comparison of different methods in an ideal case where measurement error and confounding factor do not exist and interacting cell pairs needed for Giotto and NCEM are perfectly known. (*E*) Comparison of different methods where interacting cell pairs needed for Giotto and NCEM cannot be perfectly identified. (*F*) Comparison of different methods under the existence of measurement error for cell–cell distance. (*G*) Comparison of different methods under the existence of unobtained confounding factor.

We performed extensive simulations to understand the performance of QuadST under varying parameters (e.g., quantile levels, *k*-nearest neighbors) and data sets (e.g., sample sizes) (Methods). First, we evaluated the impact of numbers of quantile levels used in QuadST on its performance (Fig. 2A). We observed that type I error rates were consistently well controlled, even if extreme quantile levels were used, such as using 999 quantile levels at 0.001, 0.002, …, 0.999 for a total of 1000 anchor cells. The study powers initially increased with the number of quantile levels but plateaued after approximately 10 quantile levels. This is mostly due to highly correlated results at additional quantile levels that contribute little to the ICG identification. These results suggest that the specification of quantile levels in QuadST need not consider error control but rather balance between study power and computation cost at a given sample size of the data. Next, we evaluated the impact of *k*-nearest neighbors used to construct an anchor–neighbor matrix in QuadST on its performance and observed a consistently well-controlled type I error rate and a slight power decrease as the number of nearest neighbors increased from one to five (Fig. 2B). Finally, to understand the performance of QuadST on small samples, we ranged sample size from 100 to 1000 and observed well-controlled type I error and steadily increased study power with sample size (Fig. 2C).

Additionally, we performed extensive simulations to compare the performance of QuadST with alternative approaches. First, we evaluated all methods in an ideal case, where cell–cell distance was perfectly measured, no unmeasured confounding factors existed in the data, and interacting versus noninteracting cell pairs could be perfectly known for use in Giotto and NCEM. All methods had well-controlled type I error, and QuadST and NCEM reached similar highest study power at 60.5% and 61.9%, followed by Giotto at 51.7% and Linear Regression (LR) at 15% (Fig. 2D). Secondly, we evaluated methods when interacting versus noninteracting cell pairs could not be perfectly identified. Giotto and NCEM were impacted, with powers reduced to 28.1% and 36.5%, whereas QuadST, not requiring this information, maintained the high power at 60.5% (Fig. 2E). Thirdly, we evaluated methods under the existence measurement errors, which biased the observed cell–cell distance. Although all methods had well-controlled type I error rate, QuadST had much higher study power at 50.9% than other methods, at 37.2% for NCEM, 28.4% for Giotto, and 14.4% for LR (Fig. 2F). Finally, we evaluated these methods under existence of unmeasured confounding factors. QuadST was the only method that can control type I error rate, albeit conservatively at 2% (Fig. 2G), whereas all others have inflations—to 16% in NCEM, 17.2% in Giotto, and 88.3% in LR. Although QuadST was overconservative, it ensured the validity of its significant identifications. Conversely, all compared methods exhibited largely inflated type I error rates, jeopardizing their validities.

### Cell–cell interaction studies in mouse cortex

**Figure 3.**
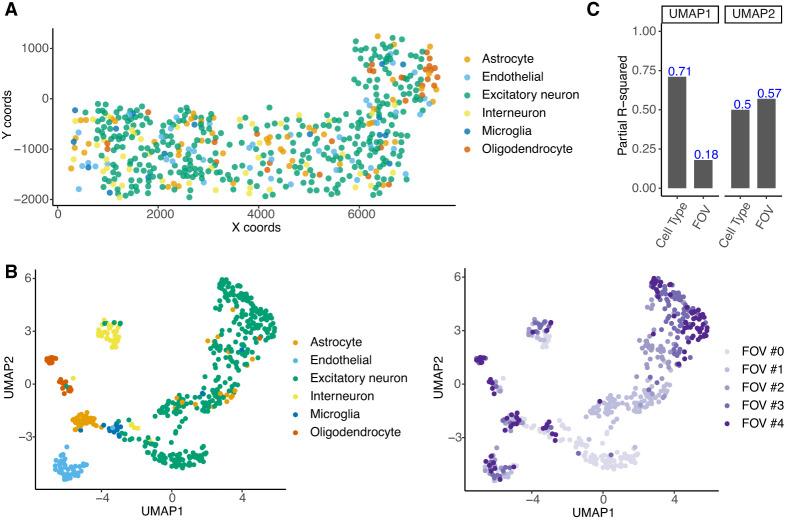
A publicly available seqFISH+ data set from mouse cortex. (*A*) Spatial map by annotated cell types in the seqFISH+ data. (*B*) UMAP of the highly variable genes in the data. (*C*) Partial R-squared explained by cell type and FOV for UMAP Dimension 1 and 2.

We first applied QuadST to study the cell–cell interaction in a mouse cortex data set profiled by the seqFISH+ technology (Eng et al. 2019; Methods). The data included expression levels of 10,000 genes from 511 cells of six cell types (Fig. 3A), imaged from five fields of view (FOVs), from a 23-day-old male mouse (C57BL/6J) cortex. The uniform manifold approximation and projection (UMAP) plot showed that cells clustered not only by cell types but also by FOVs (Fig. 3B), which was confirmed by partial *R*^2^ analysis (Fig. 3C), instructing us to adjust FOVs as covariates in the analysis.

**Figure 4.**
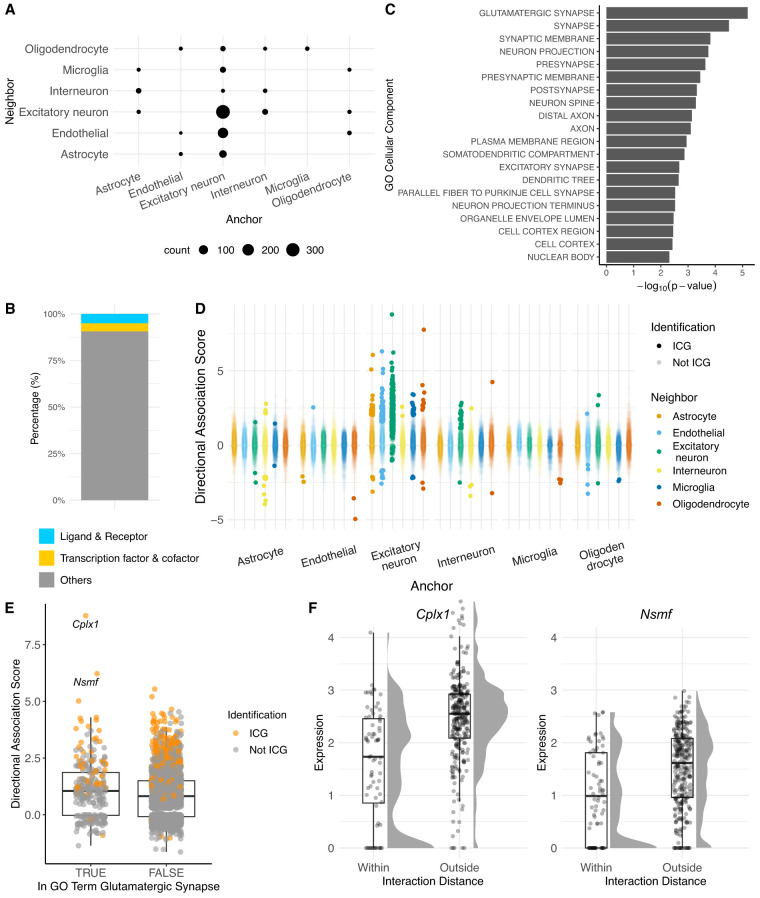
Application of QuadST to a seqFISH+ data set from mouse cortex. (*A*) Number of significant ICGs by anchor–neighbor cell-type pairs. (*B*) Functional categories of significant ICGs. (*C*) GO cellular component enrichment of ICGs in interactions among excitatory neurons. (*D*) Directional association scores of ICGs and all other genes among all anchor–neighbor cell pairs. (*E*) Box plot of directional association scores of genes in and out of the glutamatergic synapse gene set. (*F*) Distance-expression profiles of the top two ICGs.

We ran QuadST for a total of 36 anchor–neighbor cell-type pairs from six cell types on 1235–2500 highly variable genes and identified 626 ICGs across 19 out of 36 pairs at 10% FDR (Fig. 4A; Supplemental Table S1). Out of these significant ICGs, only 5.1% were ligands and receptors, and 4.4% were transcription factors and cofactors (Fig. 4B), indicating that ICGs could be genes with different functions directly or indirectly involved in cell–cell interactions. The most frequent interactions were observed among excitatory neurons, with a total of 326 significant ICGs. These ICGs were primarily involved in synapse-related functions, particularly glutamatergic synapses, as revealed by the Gene Ontology (GO) cellular component enrichment analysis (Fig. 4C). A vast majority of these ICGs (97%) displayed positive associations with cell–cell distance (Fig. 4D), further suggesting that they play crucial roles in establishing connections with distant excitatory neurons via axons and dendrites through synapses. The top two ICGs were *Cplx1* and *Nsmf* (Fig. 4E,F). *Cplx1* regulates synaptic vesicle fusion (Chang et al. 2015), and *Nsmf* facilitates the transmission of signals from both synaptic and extrasynaptic NMDA receptors to the nucleus (Gomes et al. 2023). The discoveries of these critical genes and their association patterns demonstrated the effectiveness of our method in revealing intricate details of cell–cell interactions.

**Figure 5.**
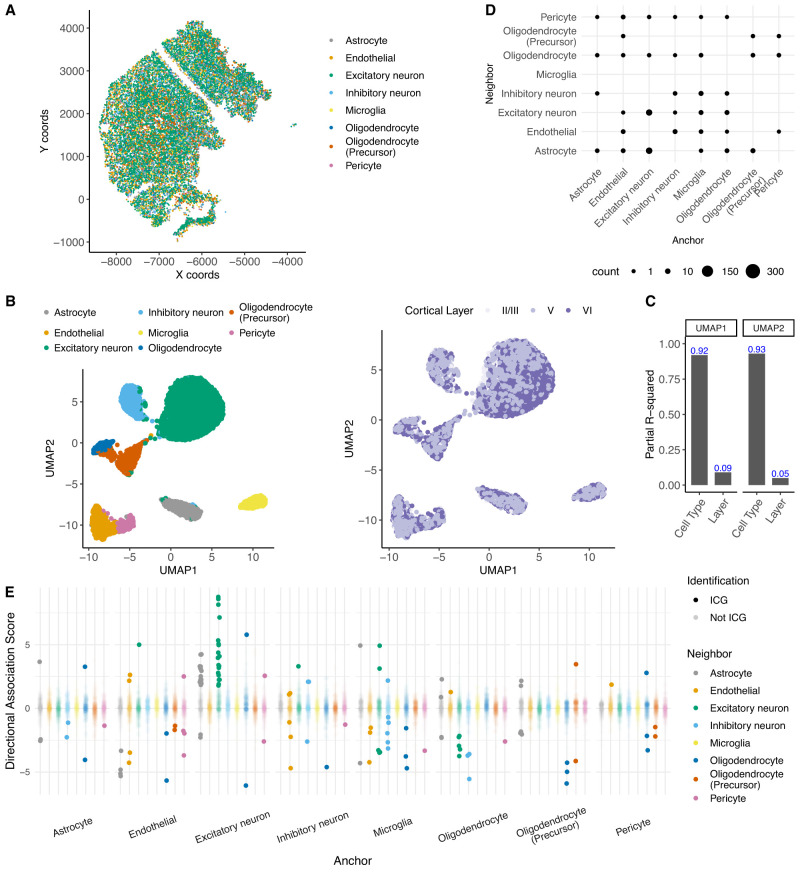
Application of QuadST to a MERFISH data set from mouse cortex. (*A*) Spatial map by annotated cell types in the MERFISH data. (*B*) UMAP by cell type and cortical layer. (*C*) Partial R-squared by cell type and cortical layer. (*D*) Number of significant ICGs by anchor–neighbor cell-type pairs. (*E*) Directional association scores of ICGs and all other genes among all anchor–neighbor cell pairs.

We next applied QuadST to a different mouse cortex data set (Allen et al. 2023) profiled by the MERFISH technology (Methods). This data set included 374 preselected genes in 13,745 cells of eight cell types from the cortical layers II–V of a 4-week-old female mouse (C57BL/6J) (Fig. 5A), and QuadST was applied to 280 highly variable genes of the cells. Cortical layers contributed slightly to variations of gene expression as demonstrated by UMAP (Fig. 5B) and partial *R*^2^ (Fig. 5C) and were included as covariates in QuadST analysis. We ran QuadST for a total of 64 anchor–neighbor cell-type pairs for the eight cell types and identified 132 significant ICGs for 36 out of 64 cell-type pairs (Fig. 5D; Supplemental Table S2). Consistent with the seqFISH+ results, interactions among excitatory neurons were most frequently identified (Fig. 5E), with all the 19 significant ICGs showing upregulated expression with cell–cell distance (Fig. 5E).

In addition to detecting cell–cell interactions and their ICGs, QuadST also provides an estimation of interaction distance specific to each cell-type pair (Supplemental Fig. S1). In these two data sets, the distances varied between 10 and 70 µm for different cell types. NCEM reported distances on the same scale but did not demonstrate variations across cell types due to their analyses of all cells collectively.

Finally, to understand how QuadST results differ from those produced by other methods, we also applied NCEM, Giotto, and LR to the seqFISH+ (Supplemental Table 3) and MERFISH (Supplemental Table 4) data. A challenging step for both NCEM and Giotto is to group cells into neighboring pairs. NCEM suggests a cutoff around 69 µm on cell–cell physical distance for grouping. Using this cutoff, NCEM identified 2450 out of 2500 highly variable genes as ICGs with interactions among excitatory neurons in seqFISH+, suggesting a potential high proportion of false discoveries. We further performed sensitivity analysis by perturbing cell–cell distance cutoffs (59 µm, 69 µm, 79 µm) and found that it was highly sensitive to the cell pair groups. For instance, NCEM detected 155 ICGs for microglia–astrocyte interactions in seqFISH+ at 59 µm but no ICGs at 69 µm. Although the distance-based cutoff is not robust, Giotto offers an alternative network-based algorithm for defining neighborhoods. However, this approach may lack sufficient resolution. For instance, in seqFISH+ data, it classified all excitatory neurons into each other's neighborhoods, failing to detect any ICGs. Finally, when applying the naive LR to the real data sets, we identified 15 ICGs out of approximately 2500 highly variable genes across all 36 cell-type pairs in seqFISH+, and 212 ICGs out of 280 genes across 64 cell-type pairs in MERFISH, yielding a MERFISH-to-seqFISH+ signal ratio of 70.98. In contrast, QuadST identified 626 ICGs in seqFISH+ and 127 in MERFISH, resulting in a ratio of 1.02. Given that MERFISH includes a large number of canonical cell markers who function distinctly from ICGs, we do not expect a high MERFISH-to-seqFISH+ signal ratio. The observed ratio in the naive LR likely reflects its inability to account for unmeasured confounders, measurement errors, or gene–gene correlations.

## Discussion

We introduce QuadST for the robust and powerful identification of cell–cell interactions and their changed genes on single-cell SRT data. QuadST effectively controls the FDRs, even under the existence of inaccurate measurements of cell–cell distances, unmeasured confounding factors, and gene–gene correlations. Simulation studies demonstrate that QuadST controls the type I error across various settings, including under the existence of unmeasured confounding factors. It also provides higher power than existing methods, especially when interacting cell pairs cannot be perfectly specified or cell–cell distance cannot be accurately measured. Applying QuadST to two data sets obtained from the mouse cortex via seqFISH+ and MERFISH technologies, we uncovered interacting cell types, genes involved in their interactions, and the corresponding distances of these interactions. We showed that our identified ICGs among excitatory neurons were enriched in synapse functions, demonstrating the effectiveness of our method in revealing intricate details of cell–cell interactions.

The attractive features of QuadST arise from the three-tiered modeling design: treating cell–cell distance as outcome, modeling its quantile levels, and contrasting signals across different quantile levels to identify ICGs. First, treating cell–cell distance as outcome reduces the influence of measurement errors on ICG identification. Current technologies often introduce significant errors when quantifying cell–cell distance. Traditional methods typically group cells into neighboring versus nonneighboring pairs based on their distances, which are largely vulnerable to these measurement errors. By treating distance as an outcome rather than a grouping variable, QuadST allows these errors to be taken in the regression error and thus limits their impact (Chen et al. 2011). Notably, QuadST employs the test statistics against the null hypothesis that gene expression and cell–cell distance are independent, which can be achieved regardless of treating distance as predictor or outcome. Second, modeling association between gene expression and cell–cell distance at different quantile levels enables QuadST to capture local signals for ICG identification. Existing methods often model two binary states, “on” and “off,” ignoring finer variations, such as stronger effects when cells are closer within the “on” state. Regressions that associate genes with the mean distance fail to capture abundant interactions that occur primarily at very short distances. By employing quantile regression, QuadST surveys the local cell–cell interaction patterns across the entire range of cell–cell distances, capturing the nuanced dynamics of cellular interactions. Lastly, contrasting signals across different quantile levels guarantees robust ICG identifications. Cellular interactions occur within complex microenvironments influenced by numerous factors, such as additional cells and extracellular elements, that may confound the analysis and cannot be fully adjusted. These factors can distort *P*-value distributions, leading to a small *P*-value under the null. Additionally, genes in cells are often correlated, causing correlated *P*-values as well. Claiming significance directly using these ill-behaved *P*-values may result in erroneous conclusions. By comparing signals across quantile levels, QuadST effectively mitigates the impact of confounding factors and gene–gene correlations, yielding reliable and accurate identifications of ICGs even in complex data sets, as statistically proved in Methods.

Successful application and interpretation of QuadST involve careful attention to several key aspects. First, QuadST performs cell-type pair–specific analysis, assuming cell types have been pre-identified. Inaccuracies in identifying cell types can reduce QuadST's study power; therefore, careful preparation work, such as data preprocessing, clustering, and annotation, is needed for high-quality analysis. Second, QuadST surveys signals at a grid of user-specified cell–cell distance quantile levels. Although it maintains well-controlled FDR even at extreme quantile levels, using too many quantile levels can reduce computational efficiency but have limited power gain. Conversely, too few quantile levels may result in missed regions of interest. To make a balance, we recommend using at least five cells between two neighboring quantile levels and a maximum of 49 quantile levels. Third, QuadST is designed to borrow information across genes for FDR-controlled identification. Technologies such as CODEX, which spatially profiles fewer than 100 proteins, might not be directly applicable by QuadST. Future studies could enhance QuadST's capabilities by integrating transcriptomics-based signals as priors. Finally, the QuadST identified ICGs are directional and not symmetric, and their interpretations cannot be reversed. For example, an ICG identified for the excitatory neuron (anchor)–astrocyte (neighbor) pair indicates this gene expressed in excitatory neurons is more confidently associated with the distance to astrocytes when the cells close together. This does not provide information on this gene's expression in astrocytes.

## Methods

### QuadST

#### Overview

QuadST is applicable to each predefined anchor–neighbor cell-type pair and consists of three key steps: (1) creating the anchor–neighbor integrated matrix; (2) modeling the association between anchor cells’ gene expression and cell–cell distance at different distance quantile levels; and (3) identifying ICGs by contrasting the signal levels between nearby and distant quantile levels (Fig. 1).

#### Anchor–neighbor integrated matrix

Suppose SRT data contain expression levels of *G* genes in *N* cells. These cells can be categorized into *K* cell types based on their expression (and sometimes spatial) profiles. For each cell-type pair (*l*, *m*), where *l*, *m* ∈ (1, …, *K*), we are interested in identifying the existence of their cell–cell interaction and ICGs. Note, when *l* ≠ *m*, we study the impact of neighboring cells in *m* cell type on gene expression of cells in *l* cell type, and when *l* = *m*, we study the impact of neighboring cells in *m* cell type on gene expression of cells in its own cell type.

The first step is to define the anchor *l*–neighbor *m* integrated matrix. We let $X_{ig}^{l}$ be the expression level of gene *g* ∈ (1, …, *G*) in each cell *i* ∈ (1, …, *N*_*l*_) in the anchor cell type *l*, where *N*_*l*_ is the number of cells for cell type *l*, $Z_{i}^{l\;}$ be the vector of cell-level covariates, and $Y_{i}^{lm}$ be the weighted averaged distance from cell *i* to its *k* nearest cells in cell type *m*, respectively. Parameter *k* denotes number of nearest neighbor cells under consideration. Let $Y_{i,t}^{lm}$ be the distance from the anchor cell *i* to its *t*^th^ nearest neighbor, then $Y_{i}^{lm}=k/\sum_{t=1}^{k}\left(1/Y_{i,t}^{lm}\right)$. When *k* = 1, $Y_{i}^{lm}$ simplifies to $Y_{i,1}^{lm}$, the distance from cell *i* to its nearest neighbor cell. Then ${\left(Y_{i}^{lm},\;X_{i1}^{l},\;\ldots ,\;X_{iG}^{l},\;Z_{i}^{l\;T}\right)}_{i=(1,\ldots ,N_{l})}$ is the anchor *l*–neighbor *m* integrated matrix. For a total of *K* cell types of interest, such matrices can be constructed for *K*^2^ anchor–neighbor cell-type pairs. As QuadST is designed to analyze on these pairs separately, we will omit the index *l* and *m*, using *Y*_*i*_, *X*_*ig*_, and ***Z***_*i*_ in the following section for simplicity.

#### Distance quantile–based model

Assuming that nearby cells are more likely to interact with each other than distant cells, with stronger interactions likely perturbing more genes, we examined the association between gene expression and cell–cell distance at different quantile levels of the distance. Unlike models focusing on mean distance, our model allows for distinct associations in nearby versus distant cells. Specifically, we considered a grid of evenly spaced quantile levels $\tau =(\tau_{1},\tau_{2},\ldots ,\tau_{J})$, such as (0.02, 0.04, …, 0.98) for 49 quantile levels, across the entire cell–cell distance distribution. At each quantile level *τ*_*j*_ ∈ (0, 1), where *j* ∈ (1, 2, …, *J*), we model the association with gene *g* as follows:

(1) $$Q_{Y_{i}}(\tau_{j}|X_{ig},Z_{i})=X_{ig}\beta_{g,\tau_{j}}+Z_{i}^{T}\alpha_{g,\tau_{j}}.$$

Here, $\beta_{g,\tau_{j}}$ captures the effects of gene *g* on the *τ*_*j*_^th^ quantile of cell–cell distance, and similarly, $\alpha_{g,\tau_{j}}$ captures the effects of cell-level covariates. The *P*-value under the null hypothesis $\beta_{g,\tau_{j}}=0$ can be obtained using a quantile rank-score test (Song et al. 2017).

It is worth noting that single-cell transcriptomics data often contain excessive zeros due to both biological and technical factors (Jiang et al. 2022). We address this issue by recognizing that the excessive zeros complement the continuous counts in providing expression abundance information and thus extend our model under zero inflation to include a nonzero indicator as an extra predictor

(2) $$Q_{Y_{i}}(\tau_{j}|X_{ig},Z_{i})=X_{ig}\beta_{g,\tau_{j}}^{\left(1\right)}+I(X_{ig}\neq 0)\beta_{g,\tau_{j}}^{\left(2\right)}+Z_{i}^{T}\alpha_{g,\tau_{j}},$$

where *I*(.) is an indicator function indicating whether gene expression is nonzero. This structure allows zero counts to have a discrete effect on the outcome from continuous values. Both $\beta_{g,\tau_{j}}^{\left(1\right)}$ and $\beta_{g,\tau_{j}}^{\left(2\right)}$ capture the effects of gene *g* on *τ*_*j*_^th^ quantile of cell–cell distance, and their consistent directions matter in interpreting the associations. Therefore, we propose to jointly test their effects while taking the directions into consideration. Specifically, we first project the gene expression predictors *A* = (*X*, *I*(X ≠ 0)) to the column space of *Z* to obtain orthogonal signals of covariates, such that $A^{*}=A-Z{\left(Z^{T}Z\right)}^{-1}Z^{T}A$, where *X* = {*X*_*ig*_} and *Z* = {*Z*_*i*_} are the matrix forms of the predictors. Then, the quantile rank score function can be defined as $S_{n}=n^{-1/2}A^{*T}\phi_{\tau }(Y-Z^{T}\hat{\alpha }),$ where *Y* = {*Y*_*i*_} is the matrix form of *Y*_*i*_, $\phi_{\tau }(u)=\tau -I(u<0)$ is an asymmetric sign function for quantile regression, and $\hat{\alpha }$ is the estimated coefficient under the null. Under the null hypothesis, *S*_*n*_ is a *p* × 1 matrix following a normal distribution with mean zero and variance $\Sigma_{n}=n^{-1}\tau (1-\tau )A^{*T}A^{*}$. We standardize *S*_*n*_ and take an average across the *p* predictors, such that $\tilde{S_{n}}=n^{-1}1^{T}\Sigma_{n}^{-1/2}S_{n}$. Then, $\tilde{S_{n}}$ is our test statistic that follows *N*(0, 1) under the null.

#### Identification of ICGs

Designed to examine associations at a grid of evenly distributed quantile levels across the entire cell–cell distance distribution, QuadST does not require knowledge of a specific distance where cell–cell interaction occurs. Specifically, from a total of *G* genes examined at a total of *J* distance quantile levels, we obtain a *G* × *J* matrix for *P*-values. Each element $p_{g,\tau_{j}}$ captures the association at the quantile level τ_*j*_ for gene *g* from Equations 1 or 2. For the *j* upper quantile levels (*J*, *J* − 1, …, *J* − *j* + 1), we define *A*_*j*_ as the *G* × *j* subset of the *P*-value matrix covering all *G* genes; similarly, for the *j* lower quantile levels (1, 2, … *j*), we define *B*_*j*_ as the *G* × *j* subset of the *P*-value matrix. Then, each element of *A*_*j*_ and *B*_*j*_, denoted as $p_{g,h}^{A_{j}}$ and $p_{g,h}^{B_{j}}$, where *h* ∈ (1, …, *j*) and *g* ∈ (1, …, *G*), captures aggregated signals from the same number of quantile levels in two tails. Then, we contrast $p_{g,h}^{A_{j}}$ and $p_{g,h}^{B_{j}}$ to identify ICGs which are more likely to be associated with cell–cell distance in nearby cells versus distant cells. Specifically, we calculate *T*_*j*_(*c*) as the ratio of the number of genes with *P*-values below a small positive cutoff *c* for the upper versus lower tails

(3) $$T_{j}(c)=\frac{\sum_{g=1}^{G}\sum_{h=1}^{j}\left(\;p_{g,h}^{A_{j}}<c\right)\;}{\sum_{g=1}^{G}\sum_{h=1}^{j}\left(\;p_{g,h}^{B_{j}}<c\right)\;}.$$

*T*_*j*_(*c*) close to 1 indicates a similar number of genes with small *P*-values for both nearby and distant cells, suggesting a lack of evidence for cell–cell interaction. Conversely, a small ratio suggests more genes with small *P*-values in nearby cells than distant cells, indicating the presence of cell–cell interactions. Therefore, an evaluation *T*_*j*_(*c*) can be used to infer the presence of cell–cell interaction and its ICGs.

It is critical to note that, under two simple assumptions, *T*_*j*_(*c*) provides an upper bound of the eFDR (Tusher et al. 2001; Efron and Tibshirani 2002). The eFDR is a data-driven, unbiased estimator of FDR that uses observed test statistics under the null hypothesis and the actual data to estimate the proportion of null hypotheses for FDR. Occasionally, eFDR exceeds 1, which can be interpreted as FDR = 1. The two simple assumptions we need are:

1. The $p_{g,h}^{A_{j}}$ and $p_{g,h}^{B_{j}}$ are symmetric under the null, such that $\#\;(\mathrm{null}\;g:\;p_{g,h}^{A_{j}}<c)=\#\;(\mathrm{null}\;g:\;p_{g,h}^{B_{j}}<c)$.
2. The $p_{g,h}^{A_{j}}$ is smaller under the alternative than under the null, resulting in $\#\;(\mathrm{null}\;g:\;p_{g,h}^{A_{j}}<c)\leq \#\;(g:\;p_{g,h}^{A_{j}}<c)$.

In this context, the eFDR can be expressed as

(4) $$eFDR_{j}(c)=\frac{\#\;(\mathrm{null}\;g:\;p_{g,h}^{B_{j}}<c\;)}{\#\;(g:\;p_{g,h}^{B_{j}}<c)}=\frac{\#\;(\mathrm{null}\;g:\;p_{g,h}^{A_{j}}<c)}{\#\;(g:\;p_{g,h}^{B_{j}}<c)}\leq \frac{\#\;(g:\;p_{g,h}^{A_{j}}<c)}{\#\;(g:\;p_{g,h}^{B_{j}}<c)}=T_{j}(c)\;$$

This feature of *T*_*j*_(*c*) is highly attractive, as it bounds the FDR without assuming well-behaved distributions of $p_{g,h}^{A_{j}}$ and $p_{g,h}^{B_{j}}$. For example, under the existence of unmeasured confounders, such as with unknown effects from extracellular factors or unknown biases from technical handling, the $p_{g,h}^{A_{j}}$ and $p_{g,h}^{B_{j}}$ can be biased to small values under the null. Similarly, under gene–gene correlation, $p_{g,h}^{A_{j}}$ and $p_{g,h}^{B_{j}}$ can have distorted distributions. In both cases, by contrasting $p_{g,h}^{A_{j}}$ and $p_{g,h}^{B_{j}}$ instead of comparing each of them to a theoretical distribution like uniform (0,1), *T*_*j*_(*c*) ensures the FDR control. Therefore, QuadST provides computationally efficient and highly robust FDR control under complex *P*-value behaviors.

To identify ICGs at a desired nominal eFDR level *α* (say 10%), we search for the *P*-value threshold *c* as

(5) $$C_{j}=\max\left\{c:\frac{\sum_{g=1}^{G}\sum_{h=1}^{j}(\;p_{g,h}^{A_{j}}<c)+d}{\sum_{g=1}^{G}\sum_{h=1}^{j}(\;p_{g,h}^{B_{j}}<c)+d}\leq \alpha \right\},$$

where *d* > 0 is a small constant to make eFDR control conservative. Finding the appropriate threshold **C**_*j*_ indicates that cell–cell interaction exists at or below the desired nominal FDR level *α* at the quantile level *τ*_*j*_. In other words, if **C**_*j*_ cannot be found, we claim that there is no cell–cell interaction in this cell-type pair. Under the existence of **C**_*j*_, the significant genes ${\tilde{G}}_{j}\;$ are the genes with eFDR < *α*.

ICGs are the significant genes at quantile level *I*, that is, $ICG={\tilde{G}}_{I}$, where *I* maximizes the number of identified genes, that is, $I=(\;j:\max(\#{\tilde{G}}_{j}))$. Meanwhile, the cell–cell interaction distance is the distance corresponding to quantile level *I*.

### Simulation studies

#### Simulation settings

We have performed comprehensive simulations to evaluate the performance of QuadST, including evaluating the impact of quantile levels, number of nearest neighbors (*k*), and number of cells (sample size). We also compared the results with three alternative methods, Giotto, NCEM, and LR, that identify genes involved in cell type–specific cell–cell interactions. Here, we first describe a basic simulation set up that serves as the skeleton of all simulations, as well as the general implementation of QuadST and comparison methods, and then explain the alternations in each individual simulation study.

##### Basic simulation setup (skeleton)

We randomly generated 5000 cells uniformly distributed in a unit square. These cells were equally divided into five cell types, with one cell type randomly chosen as the anchor cell type and another cell type randomly selected as the neighbor cell type. In each anchor cell, we simulated 2000 genes whose expression levels were block-wise correlated, with a block size of 100. Within each block, gene expression levels followed a multivariate normal distribution with an autocorrelation coefficient 0.7 and homogeneous variance. We also assumed that 10% of the anchor cells, which were most proximal to cells in the neighbor cell type, were involved in cell–cell interactions, which perturbed the expression levels of 20% genes (i.e., four blocks out of 20 blocks) in these cells. For genes not involved in cell–cell interactions, their expression levels had a mean 0 and variance 1. For the four blocks of genes involved in cell–cell interactions, we simulated four different distance-expression association patterns as follows:

*Block 1: Constant Mean Shift.* This pattern represented an “on/off” pattern, where cell–cell interaction was turned “off” above the 10% cell distance threshold and “on” below the threshold. Under the interaction, the effect was a constant mean shift from 0 to $\mu_{\delta }$, where $\mu_{\delta }∼N(0.4,\;0.2)$.
*Block 2: Dosage Mean Shift.* This pattern represented a “dosage” pattern, where cell–cell interaction increased as cells become closer after being turned “on” at the distance threshold. The mean shift was generated as a piecewise linear function of distance (*Y*), such that $\mu_{\delta }(Y)=a(Y-d_{c})I(Y<d_{c})$, where *d*_*c*_ was the distance cutoff for 10% of cells and *a* ∼ *N*( − 150, 75) was the slope for dosage effect.
*Block 3: Constant Mean Shift with Variance Increase.* Cell–cell interaction not only impacted the mean in the “on/off” pattern as in Block 1, but also impacted the variance of gene expression from 1 to $var_{\delta }=2$.
*Block 4: Dosage Mean Shift with Variance Increase.* Cell–cell interaction not only impacted the mean in the “dosage” pattern as in Block 2 but also the variance of gene expression from 1 to $var_{\delta }=2$.

##### Implementation of QuadST and comparison methods

We compared QuadST with the three other methods, Giotto, NCEM, and LR, in simulation studies. Unless revised in the individual settings, we used one nearest neighbor and 49 quantile levels in QuadST analysis. Both Giotto and NCEM analysis required the determination of interacting versus noninteracting cell pairs, such as by using cell–cell distance or network. These interacting cell pairs can hardly be well estimated in real data, but in simulations, we used the simulated known ground truth to demonstrate their best performances. Finally, we developed a LR approach to demonstrate the importance of modeling and contrasting different quantile levels, as designed in QuadST, rather than focusing on mean distance. This method modeled the mean cell–cell distance on gene expression level using linear regression, as such $E[Y_{i}]=X_{ig}\beta_{g}+Z_{i}^{T}\alpha_{g}$. ICGs in all methods were all claimed significant at 10% FDR. Averaged results from 10 times Monte Carlo simulations were reported.

*Individual Setting 1* (relevant to Fig. 2A). To evaluate the impact of number of quantile levels used in QuadST on its performance, we performed simulations using the basic simulation setup and evaluated QuadST that uses six different sets of evenly distributed quantile levels, including three quantile levels at 0.25, 0.5, 0.75, four quantile levels at 0.2, 0.4, 0.6, 0.8, nine quantile levels at 0.1, 0.2, …, 0.9, 19 quantile levels at 0.05, 0.1, …, 0.95, 49 quantile levels at 0.02, 0.04, …, 0.98, 99 quantile levels at 0.01, 0.02, …, 0.99, and 999 quantile levels at 0.001, 0.002, …., 0.999. In our simulated data with 1000 cells, specifying 999 quantile levels provides assessment of QuadST under very extreme tails.

*Individual Setting 2* (relevant to Fig. 2B). To evaluate the impact of using a different number of *k* in *k*-nearest neighbors in QuadST on its performance, we performed simulations also using the basic simulation setup and evaluated QuadST using five *k* values at 1, 2, …, 5.

*Individual Setting 3* (relevant to Fig. 2C). To evaluate the impact of small sample size on the performance of QuadST, instead of simulating 1000 cells per cell type as in the basic simulation setup, we also simulated 100, 300, 500, and 700 cells per cell type.

*Individual Setting 4* (relevant to Fig. 2D). We evaluate the FDR and power of QuadST and other methods in the ideal case using the basic simulation setup.

*Individual Setting* 5 (relevant to Fig. 2E). As both Giotto and NCEM required the determination of interacting versus noninteracting cell pairs, we compared QuadST and LR to these two methods using a distance threshold that was 1.4 times the simulated value, approximately doubling the interacting cell pairs from the simulated values. This demonstrated the performance of these methods when interacting cell pairs could not be estimated with accuracy.

*Individual Setting 6* (relevant to Fig. 2F). To evaluate the robustness of these methods, under the biasedly quantified cell–cell distance (*Y*), we specified that observed distance $\tilde{Y}=Y+\mathrm{uniform}(0,\;0.005)$, and evaluated their performance under observed $\tilde{Y}$.

*Individual Setting 7* (relevant to Fig. 2G). To understand the impact of unmeasured confounding factors on analysis, we introduced region as a factor impacting cell locations and expression levels. We split the unit square into four equal-sized regions (*R*_1_ − *R*_4_), altered the locations of neighboring cells to appear in *R*_1_ − *R*_3_, and increased by 0.2 the mean expression level for all genes in anchor cells in *R*_4_. In this case, both cell composition and gene expression level varied by region, making it a confounding factor for the association between cell–cell distance and expression. We assumed region information was not accessible to us and compared QuadST with other methods under the unmeasured confounder.

### Data analysis

#### seqFISH+ data set

High-throughput single-cell SRT data obtained from the cortex of a 23-day-old male mouse (C57BL/6J) using seqFISH+ were downloaded for analysis (Eng et al. 2019). It included spatial map and expression levels for 10,000 genes in 523 cells, imaged from five fields of view. The cells were categorized by the original study into nine major cell types, including excitatory neuron (n = 325), interneuron (n = 42), astrocyte (n = 54), endothelial (n = 45), oligodendrocyte (n = 29), microglia (n = 16), neural stem cells (n = 6), neuroblast (n = 5), and ependymal (n = 1).

#### MERFISH data set

We also analyzed the single-cell SRT data from mouse frontal cortex, profiled using MERFISH (Allen et al. 2023). The analysis focused on a 4-week-old female mouse (C57BL/6J) similar in age to seqFISH+ data. The data consists of gene expression levels for 374 genes in 13,745 cells collected from cortical layers II–V. These cells have been categorized into eight major cell types: excitatory neuron (n = 7129), inhibitory neuron (n = 1161), astrocyte (n = 1333), microglia (n = 787), oligodendrocyte (n = 1153), oligodendrocyte-precursor (n = 462), endothelial (n = 1360), and pericyte (n = 360). The spatial coordinates of cells were provided in CELLxGENE repository (shown in the key resource table of the reference Allen et al. 2023).

#### Data set preprocessing

To preprocess seqFISH+ data, we stitched the cell's coordinates obtained from five FOVs to provide the global cell spatial coordinates. We required a minimum of 10 cells for QuadST analysis and thus focused our analyses on six cell types. Then, we normalized gene counts across all remaining cells using scran (Lun et al. 2016), which adjusted for cell-specific bias due to cell-to-cell difference in library size and capture efficiency. Following the practice in Eng et al. (2019), our analysis focused on highly expressed genes whose expression levels belong to top 25% quantile in each cell type. Then, the cell-specific bias adjusted counts were transformed to normal distributions by (1) calculating the empirical cumulative distribution function, (2) using an inverse cumulative distribution function to transform it into standard normal distribution, and (3) shifting the minimum to 0. Similarly, in the preprocess of MERFISH data, we normalized gene counts using scran to adjust for cell-specific bias, kept genes whose expression levels belong to top 75% quantile in each cell type, and transferred the counts into normal distributions.

#### UMAP and covariate effect analysis

In each SRT data set, we performed UMAP analysis on gene expression data using the R package ‘umap’ (https://CRAN.R-project.org/package=umap; McInnes et al. 2018). Then, we calculated the partial *R*^2^ of each covariate on UMAP coordinates using a generalized linear model using the R package ‘rsq’ (https://CRAN.R-project.org/package=rsq), where each UMAP coordinate was considered as a response and covariates of interest were considered as predictors. The R version used in this study was 4.4.1 (R Core Team 2024).

#### QuadST analysis in seqFISH+ data set

We ran QuadST on the preprocessed seqFISH+ data set. For a total of six cell types annotated by the source, we created a total of 36 (6 × 6) anchor–neighbor integrated matrices for all pairs of the same and different cell types. Depending on the cell type, a total of 1235–2500 highly variable genes were considered for analysis, and fields of view were included as covariates. Given that excessive zeros were observed in these genes, we used the distance quantile-based model given by Equation 2 to test distance-expression association at the evenly spaced distance quantile levels. For cell types with a relatively large sample size, such as excitatory neuron (n = 325), we chose a maximum number of 49 quantile levels at 0.02, 0.04, …, 0.98 for analysis. For cell types that had few cells, we chose the number of quantile levels to ensure a minimum of five cells between two adjacent quantile levels. For example, for microglia (n = 16), we used only three quantile levels, at 0.25, 0.5, 0.75. We contrasted the resulting *P*-values at different quantile levels as described in QuadST to identify ICGs for each cell-type pair at 10% FDR. To determine the association direction and understand the association confidence, we extracted the combined *Z*-score and derived the directional association score as the sign(*Z*-score)⋅($-\log_{10}P\text{-}\mathrm{value}$) at the interaction quantile level (I).

#### QuadST analysis in MERFISH data set

Similar to seqFISH+ analysis, we applied QuadST to the preprocessed MERFISH data set for a total of eight cell types annotated by the source. We analyzed the gene expression levels of 280 highly variable genes for 64 cell-type pairs, adjusting cortical layers as covariates. The distance quantile-based model for zero-inflated genes was also used in these data to test the association. Given the large sample size for all cell types, 49 quantile levels at 0.02, 0.04, …, 0.98 were used for analysis. ICGs were identified at 10% FDR.

#### ICG function annotation using existing databases

To examine known biological roles of ICGs identified in the seqFISH+ data set, we looked up existing databases on ligands and receptors (Shao et al. 2021) as well as transcription factors and cofactors (Zhou et al. 2017). We obtained a set of mouse ligands and receptors from CellTalkDB (Shao et al. 2021) (mouse_lr_pair.txt; http://tcm.zju.edu.cn/celltalkdb/). We obtained the mouse transcription factors and cofactors from a supplemental table (41467_2017_BFncomms15089_MOESM3449_ESM.xlsx) in Zhou et al. (2017).

#### GO cellular component enrichment analysis

To test the GO cellular component terms enriched for ICGs among excitatory neurons, we performed the Wilcoxon rank-sum test comparing *P*-values from genes overlapping versus nonoverlapping with a given GO cellular component gene set. The reported *P*-values were adjusted for multiple testing (i.e., FDR 10%). The GO cellular component gene sets for mouse (m5.go.cc.v2023.1.Mm.symbols.gmt) were downloaded from the GSEA website (https://www.gsea-msigdb.org/gsea/msigdb/mouse/collections.jsp).

#### Analyses with comparison methods in seqFISH+ and MERFISH data sets

To understand how QuadST produces different results from the comparison methods, we also applied NCEM, Giotto, and LR to analyze the seqFISH+ and MERFISH data. To implement Giotto, we first built a spatial network based on the Delaunay triangulation, as suggested by the method, and identified cells with a common edge in the network as neighbors and cells that are not indirectly connected as nonneighbors. Then, we compared expression levels of the highly variable genes separately for each cell-type pair between neighboring with nonneighboring cell pairs. To control for the covariate effects from the FOVs in seqFISH+ and cortex layers in MERFISH within the association, we revised the Giotto pipeline by using regression instead of *t*-test. To implement NCEM, which suggested an average 69 µm cell–cell interacting distance on multiple platforms including MERFISH, we applied three distance cutoffs (59 µm, 69 µm, and 79 µm), controlling for the same covariates as above. Notably, as NCEM performs analysis using all cells instead of by cell-type pairs, we analyzed the 2500 highly variable genes in seqFISH+ identified across all cells, rather than by cell types as done in QuadST and Giotto, as well as the same 280 genes in MERFISH. In LR, we regressed cell–cell distance on expression, controlling FOVs in seqFISH+ and cortex layers in MERFISH as covariates. In all analyses, we controlled for 10% FDR for significance calling.

### Software availability

QuadST, together with source codes for reproducing all the analyses, is available at GitHub (https://github.com/songxiaoyu/QuadST) and as Supplemental Code.

## Supplemental Material

Supplement 1

Supplement 2

Supplement 3

Supplement 4

Supplement 5

Supplement 6
