# Supplementary material for: QuadST identifies cell–cell interaction–changed genes in spatially resolved transcriptomics data
Source: Genome Res. 2025 Aug;35(8):1821–31. doi: 10.1101/gr.279859.124 (PMC12315707; doi:10.1101/gr.279859.124)
Supplement: Supplement 1 [file Supplemental_Figure_S1.pdf]

Supplemental materials for QuadST identifies cell–cell interaction-changed genes in spatially resolved transcriptomics data

Supplemental Figure

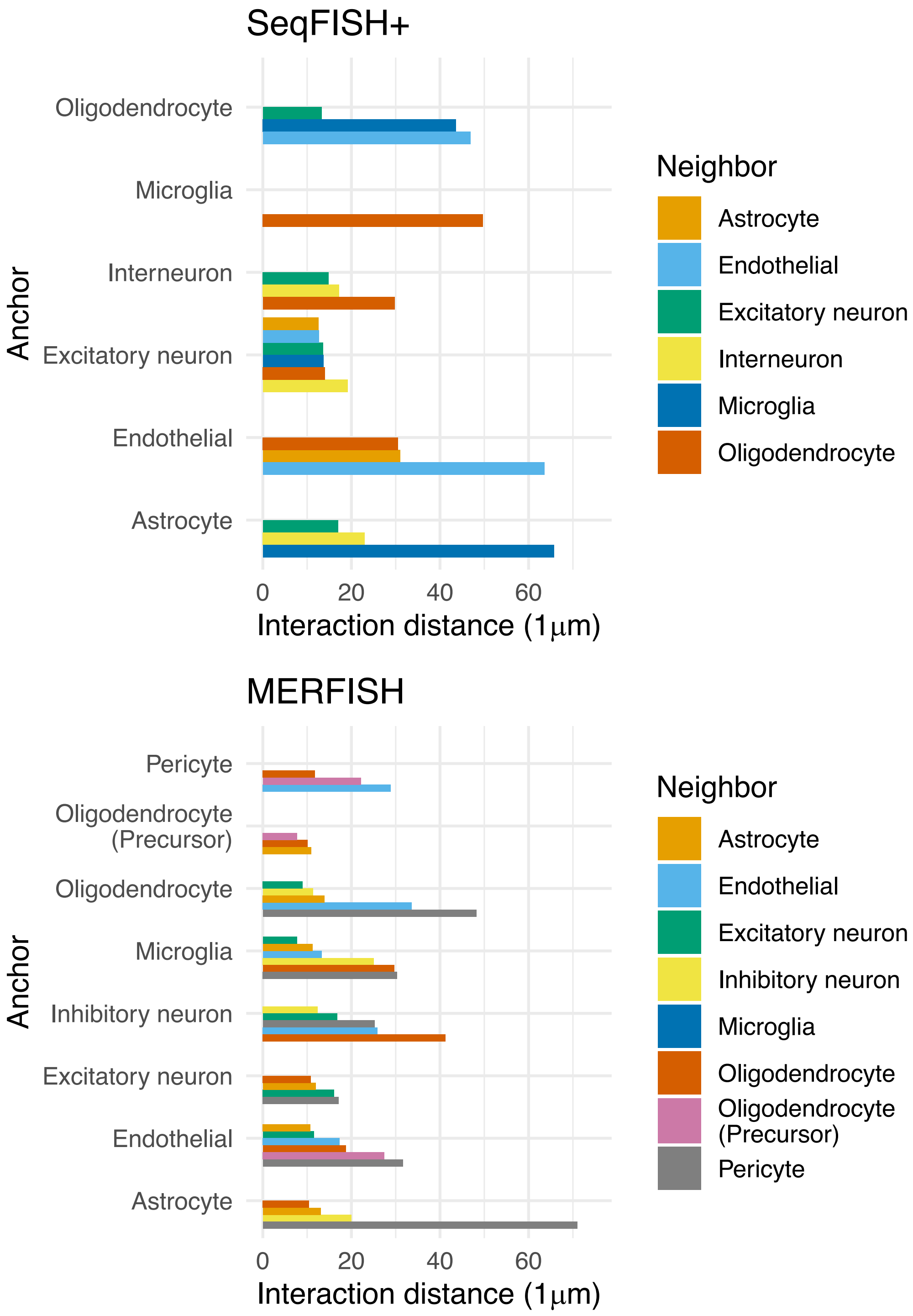

Supplementary Figure S1. Cell-type–specific interaction distance estimation by QuadST
